# TNF-α induces vascular insulin resistance via positive modulation of PTEN and decreased Akt/eNOS/NO signaling in high fat diet-fed mice

**DOI:** 10.1186/s12933-016-0443-0

**Published:** 2016-08-25

**Authors:** Rafael Menezes da Costa, Karla Bianca Neves, Fabíola Leslie Mestriner, Paulo Louzada-Junior, Thiago Bruder-Nascimento, Rita C. Tostes

**Affiliations:** 1 Department of Pharmacology, Ribeirao Preto Medical School, University of Sao Paulo, Ribeirao Preto, SP Brazil; 2 Division of Clinical Immunology, Department of Clinical Medicine, Ribeirao Preto Medical School, University of Sao Paulo, Ribeirao Preto, SP Brazil

**Keywords:** High fat diet, Insulin, TNF-α, PTEN, Vascular function

## Abstract

**Background:**

High fat diet (HFD) induces insulin resistance in various tissues, including the vasculature. HFD also increases plasma levels of TNF-α, a cytokine that contributes to insulin resistance and vascular dysfunction. Considering that the enzyme phosphatase and tension homologue (PTEN), whose expression is increased by TNF-α, reduces Akt signaling and, consequently, nitric oxide (NO) production, we hypothesized that PTEN contributes to TNF-α-mediated vascular resistance to insulin induced by HFD. Mechanisms underlying PTEN effects were determined.

**Methods:**

Mesenteric vascular beds were isolated from C57Bl/6J and TNF-α KO mice submitted to control or HFD diet for 18 weeks to assess molecular mechanisms by which TNF-α and PTEN contribute to vascular dysfunction.

**Results:**

Vasodilation in response to insulin was decreased in HFD-fed mice and in ex vivo control arteries incubated with TNF-α. TNF-α receptors deficiency and TNF-α blockade with infliximab abolished the effects of HFD and TNF-α on insulin-induced vasodilation. PTEN vascular expression (total and phosphorylated isoforms) was increased in HFD-fed mice. Treatment with a PTEN inhibitor improved insulin-induced vasodilation in HFD-fed mice. TNF-α receptor deletion restored PTEN expression/activity and Akt/eNOS/NO signaling in HFD-fed mice.

**Conclusion:**

TNF-α induces vascular insulin resistance by mechanisms that involve positive modulation of PTEN and inhibition of Akt/eNOS/NO signaling. Our findings highlight TNF-α and PTEN as potential targets to limit insulin resistance and vascular complications associated with obesity-related conditions.

## Background

Obesity is an important cause of morbidity and mortality worldwide [1, 2]. Overweight and obesity trigger metabolic abnormalities such as dyslipidemia, insulin resistance and vascular dysfunction, which contribute to the development of type 2 diabetes and cardiovascular diseases [3].

Insulin plays an important role on vascular tone control [4–6]. Insulin binding to the insulin receptor (IR) activates the phosphatidylinositol 3-kinase (PI3K)/Akt signaling pathway, resulting in endothelial nitric oxide synthase (eNOS) activation, nitric oxide (NO) release and vasodilation [7, 8]. Vascular insulin resistance is considered a primary defect in vascular dysfunction [9], and inflammatory mediators are potential contributors to insulin resistance [10].

Obesity is often associated with resistance to vascular actions of insulin [11]. Obesity is also tightly related to high levels of inflammatory mediators, including the cytokine tumor necrosis factor-alpha (TNF-α) [12]. TNF-α content is increased in murine adipose tissue, and increased circulating TNF-α levels are reported in obese humans and experimental animal models of obesity [13].

It is well established that TNF-α induces insulin resistance [14, 15]. In the vasculature TNF-α reduces IRS-1 phosphorylation and decreases NO release by the PI3K/Akt/eNOS pathway [16]. Moreover, blockade of TNF-α in obese rats increases vascular sensitivity to insulin and mice lacking TNF-α receptors remain insulin sensitive when submitted to high-fat diet (HFD) [17].

Although various studies demonstrated a crosstalk between TNF-α and insulin resistance, the mechanisms involved remain to be elucidated. Preliminary evidence indicates that increased levels of the enzyme phosphatase and tensin homologue (PTEN), widely implicated as a negative regulator of insulin/Akt signaling [18], negatively affect insulin sensitivity [19]. A recent study showed that PTEN haploinsufficiency is a monogenic cause of profound constitutive insulin sensitization. Moreover, PTEN mutations increase risks of obesity and cancer but decreases risk of type 2 diabetes [20], showing that in fact proteins related to metabolism and cell growth are closely associated with the development of metabolic diseases.

TNF-α is closely linked to PTEN regulation [21]. In human leukemic cells TNF-α increases PTEN protein expression via various nuclear transcription factors [22]. In addition, TNF-α increases PTEN phosphorylation in C2C12 cells, a mouse myoblast cell line, leading to insulin resistance [23]. Selective PTEN deletion in skeletal muscle protects against the development of fat- and age-dependent insulin resistance [24]. However, it is not known whether a similar mechanism takes place in the vasculature or whether such mechanism contributes to insulin resistance associated with HFD/obesity.

Therefore, the present study tested the hypothesis that HFD induces vascular insulin resistance via increased PTEN activity and impaired Akt/eNOS signaling. In addition, we investigated whether TNF-α triggers PTEN-mediated vascular insulin resistance in HFD-fed animals.

## Methods

### Animals and diets

All experimental protocols were performed in accordance with the Ethical Principles in Animal Experimentation approved by the Brazilian College of Animal Experimentation (COBEA) and were approved by the Ethics Committee on Animal Use (CEUA) of the University of Sao Paulo, Ribeirao Preto Campus, Brazil (Protocol no 149/2014).

Five week-old male C57Bl/6J and TNF-α receptor-deficient mice (TNF-α KO) were obtained from the Laboratory of Molecular Immunology and Embryology, Transgenose Institute, Centre National de la Recherche Scientifique (CNRS), Orléans, France and maintained in the Animal Facility of the University of Sao Paulo, Ribeirao Preto, Brazil on 12-h light/dark cycles under controlled temperature (22 ± 1 °C) with ad libitum access to food and water. After a one-week acclimatization period, mice were divided into 2 groups: (1) mice maintained in control diet (protein 22 %, carbohydrate 70 % and fat 8 % of energy, PragSolucoes); (2) mice receiving HFD (protein 10 %, carbohydrate 25 % and fat 65 % of energy, PragSolucoes) for 18 weeks. After the treatment period, mice were killed by carbon dioxide (CO_2_) inhalation.

### Nutritional and metabolic profile of high fat diet-induced obese mice

Nutritional profile was weekly determined by analyzing the caloric intake, feed efficiency, body weight and body fat. Caloric intake (per mouse) was calculated by the weekly food intake multiplied by the dietary energetic value. Feeding efficiency, defined by the ability to transform consumed calories into body weight, was determined with the formula: mean body weight gain (g)/total calorie intake. Animal body weight was measured weekly and obesity was defined using the adiposity index ([body fat (g)/final body weight (g)] × 100). Body fat was calculated by summing the epididymal, retroperitoneal and visceral fat [25]. After 18 weeks of HFD, glucose concentrations were determined in serum samples from mice fasted for 12 h, using an enzymatic colorimetric glucose oxidase method (Doles®). Plasma insulin concentration (ng/mL) was determined by radioimmunoassay (Insulin Kit®). Insulin sensitivity was calculated using the HOMA-IR index (Homeostasis Model Assessment) [26], which takes into account insulin and fasting blood glucose levels, using the following mathematical formula: HOMA-IR = fasting insulin × fasting glucose/22.5.

### Oral glucose tolerance test

The oral glucose tolerance test (OGTT) was performed to evaluate glucose tolerance. Mice were deprived of food for 6 h. Blood was sampled from the caudal vein immediately before (baseline, *t*_*0*_) and after (*t*_*15*_*, t*_*30*_*, t*_*60*_*, t*_*90*_*, t*_*120*_ min) administration of 2 g of glucose/kg by oral gavage. Glucose levels were determined using a glucose analyzer (Accu-Check, Roche Diagnostics).

### Assessment of vascular function

Mesenteric vascular beds were isolated from C57Bl/6J and TNF-α KO mice fed with control diet or HFD. Second-order branches of the superior mesenteric artery were dissected and mounted on a wire myograph (DMT, Danish Myo Technology, Aarhus, Denmark). Vessel segments (2 mm in length) were mounted on 25 µm wires in a vessel bath chamber for isometric tension recording and equilibrated for 30 min in Krebs-Henseleit-modified physiological salt solution (120 mM NaCl, 25 mM NaHCO_3_, 4.7 mM KCl, 1.18 mM KH_2_PO_4_, 1.18 mM MgSO_4_, 2.5 mM CaCl_2_, 0.026 mM EDTA, and 5.5 mM glucose), at 37 °C, continuously bubbled with 95 % O_2_ and 5 % CO_2_, pH 7.4. At the beginning of each experiment, arteries were contracted with KCl 120 mM to test for functional integrity. Endothelial function was assessed by testing the relaxant effect of acetylcholine (ACh, 2 µM) on vessels contracted with phenylephrine (PE, 2 µM). Rings exhibiting a vasodilator response to ACh greater than 80 % were considered endothelium-intact vessels. Concentration–response curves to Insulin (10^−10^–10^−5^ M) were performed in endothelium-intact arteries to assess insulin-dependent relaxation. In some protocols, arteries were pre-incubated with a vanadium complex that acts as a highly potent and specific phosphorylation inhibitor of PTEN [27] (VO-OHpic, 10^−4^ M), TNF-α (5 ng/mL), TNF-α inhibitor (Infliximab, 10^−6^ M), Akt activator (YS-49, 10^−6^ M) or eNOS inhibitor (L-NAME, 10^−5^ M), 60 min prior to the concentration–response curves.

### Nitric oxide metabolites levels

The mesenteric bed, free from adipose tissue, was immediately frozen in liquid nitrogen, pulverized and homogenized in 20 mM Tris–HCl (pH 7.4). The samples were centrifuged (5000×*g*, 10 min, 4 °C) and the total protein content was quantified using the Bradford method (Bio-Rad) [28]. The samples were analyzed in duplicate for nitrite and nitrate (NOx) using chemiluminescence-based assay ozone. Briefly, mesenteric bed samples were treated with cold ethanol (1:2 mesenteric bed to ethanol, for 30 min at −20 °C) and centrifuged (4000×*g*, 10 min). NOx levels were measured by injecting 25 μL of supernatant in a container vent glass containing 0.8 % of Vanadium (III) in HCl (1 N) at 90 °C, which reduces NOx into NO gas. A stream of nitrogen was bubbled through the purge vessel containing vanadium (III) with sodium hydroxide [NaOH (1 N)], and then through an analyzer (Sievers Nitric Oxide Analyzer® 280, GE Analytical Instruments, Boulder, CO, USA).

### Fluorescence detection of nitric oxide production

NO production was determined by the fluorescent NO indicator, 5,6 Diaminofluorescein diacetate (DAF-2 DA). Mesenteric arteries were embedded in Tissue Tek® O.C.T. Compound (Sakura Finetek, Torrance, CA, USA). Unfixed frozen cross Sects. (5 μm) were incubated with DAF-2 DA (12.5 μM; Sigma) diluted in phosphate buffer with CaCl_2_ (0.4 mM) and insulin (2 µM); in a light protected and humidified chamber at 37 °C for 1 h. Fluorescence was detected with a 490–515 nm long-pass filter, under a microscope (Olympus, BX50) with a 100× objective lens coupled to a digital camera. Fluorescent images were analyzed by measuring the mean optical density of the fluorescence in a computer system (Image J software) and normalized by the area.

### Western Blot analysis

Mesenteric vascular beds were frozen in liquid nitrogen and homogenized in a buffer (50 mM Tris/HCl, 150 mM NaCl, 1 % Nonidet P40, 1 mM EDTA, 1 μg/ml leupeptin, 1 μg/ml pepstatin, 1 μg/ml aprotinin, 1 mM sodium orthovanadate, 1 mM PMSF and 1 mM sodium fluoride). Proteins were extracted (60 μg) and separated by electrophoresis on 10 % polyacrylamide gel, and transferred on to nitrocellulose membranes. Non-specific binding sites were blocked with 5 % BSA in TBS containing 0.1 % Tween 20 (for 1 h at 24 °C). Membranes were incubated with antibodies (at the indicated dilutions) overnight at 4 °C. Antibodies were used as follows: Phospho-Akt^(Ser473)^ (1:1000 dilution; Cell Signaling Technology), Phospho-eNOS^(Ser1177)^ (1:500 dilution; Cell Signaling Technology), Phospho-eNOS^(Thr495)^ (1:500 dilution; Cell Signaling Technology), PTEN (1:500 dilution; Cell Signaling Technology), Phospho-PTEN (1:500 dilution; Cell Signaling Technology) and anti-β-actin (1:3000 dilution; Abcam). After incubation with secondary antibodies, signals were obtained by chemiluminescence, visualized by autoradiography and quantified densitometrically.

### Plasma TNF-α level

Plasma TNF-α concentration was measured with mTNF-alpha DuoSet ELISA assay kit (DY410-R&D Systems, USA).

### Compounds

Phenylephrine, acetylcholine, L-NAME, VO-OHpic and YS-49 were purchased from Sigma Chemical Co (St. Louis, MO, USA). Insulin (Insunorm®) was purchased from Aspen Pharma. Infliximab (Remicade®) was purchased from Janssen Biologics.

### Data and statistical analyses

Relaxation responses to Insulin are expressed as a percentage of contraction in response to PE. The individual concentration–response curves were fitted into a curve by non-linear regression analysis. *p*D_2_ (defined as the negative logarithm of the EC_50_ values) and maximal response (Emax) values were compared by Two-way analysis of variance (ANOVA) followed by the Bonferroni post hoc test. The Prism software, version 5.0 (GraphPad Software Inc., San. Diego, CA, USA) was used to analyze these parameters as well as to fit the sigmoidal curves. Data are presented as mean ± SEM. N represents the number of animals used *p* values less than 0.05 were considered significant.

## Results

### Metabolic parameters in C57Bl/6J and TNF-α KO mice fed with control and high-fat diets

**Fig. 1:**
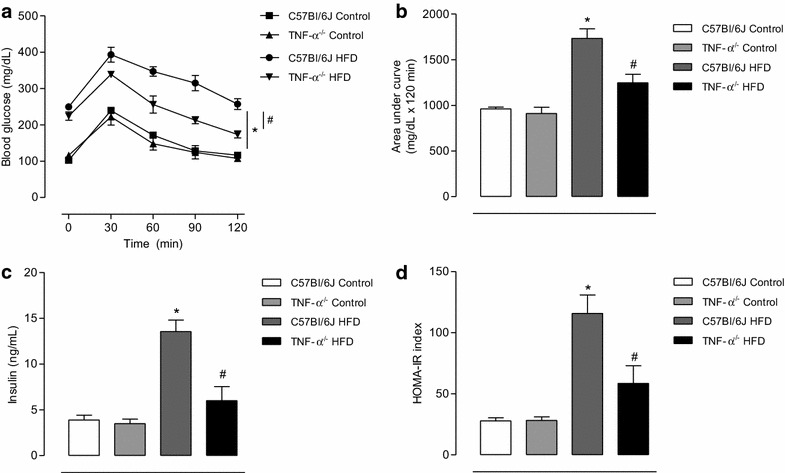
TNF-α contributes to glucose intolerance and increased insulin levels in HFD-fed mice. OGTT was performed in C57Bl/6J and TNF-α KO mice fed with control or HFD diets (for 18 weeks). After a 6 h-fasting period, baseline blood glucose was measured. Mice received 2 mg/kg glucose by gavage and blood samples were collected at 30, 60, 90 and 120 min after the challenge (**a**). Area under the curve (AUC) in the plot of blood glucose concentration against time (**b**). Insulin plasma levels (**c**). HOMA-IR index (**d**). Results represent the mean ± S.E.M. n = 7–8 in each experimental group. *p < 0.05 vs. C57Bl/6J Control, ^#^p < 0.05 vs. C57Bl/6J HFD

After 18 weeks on the HFD there was a marked increase in all nutritional and anthropometric parameters both in C57Bl/6J mice and in TNF-α KO mice (Table 1) compared with animals on the control diet. No difference in glucose tolerance, determined by the OGTT, was observed between C57Bl/6J mice and TNF-α KO mice fed with control diet. HFD decreased glucose tolerance in C57Bl/6J, whereas TNF-α deletion partially protected from HFD-induced glucose intolerance (Fig. 1a, b). In addition, insulin plasma levels and HOMA-IR index were increased in HFD-fed C57Bl/6J mice compared with their control mice. TNF-α deficiency partially prevented the increase in insulin plasma levels and HOMA-IR index (Fig. 1c, d).

**Table 1:** Characteristics of C57Bl/6J and TNF-α receptors deficient mice fed with control and high fat diets

	Control diet	Control diet	High fat diet	High fat diet
	C57Bl/6J	TNF-α KO	C57Bl/6J	TNF-α KO
Initial body mass (g)	20.9 ± 0.5	20.6 ± 0.3	21.7 ± 0.4	21.2 ± 0.4
Final body mass (g)	28.8 ± 0.6	26.6 ± 0.6	42.5 ± 0.8*	40.9 ± 0.9*
Caloric intake (kcal/week)	74.8 ± 0.5	74.2 ± 0.5	91.4 ± 1.0*	94.8 ± 0.8*
Weight gain (g)	7.9 ± 0.4	5.9 ± 0.3	20.8 ± 0.9*	18.8 ± 1.1*
Feed efficiency (g/kcal) ×100	0.3 ± 0.04	0.2 ± 0.04	0.8 ± 0.08*	0.8 ± 0.03*
Epididymal fat (g)	0.50 ± 0.02	0.47 ± 0.03	4.41 ± 0.07*	4.13 ± 0.07*
Visceral fat (g)	0.15 ± 0.02	0.12 ± 0.02	2.85 ± 0.03*	2.77 ± 0.04*
Retroperitoneal fat (g)	0.14 ± 0.07	0.15 ± 0.03	2.99 ± 0.03*	1.78 ± 0.04*
Total fat (g)	0.79 ± 0.05	0.77 ± 0.09	10.25 ± 0.11*	8.72 ± 0.21*
Adiposity index (%)	2.24 ± 0.1	1.77 ± 0.2	13.27 ± 0.6*	12.25 ± 0.7*
Glycemia (mg/dL)	100.1 ± 2.4	96.8 ± 3.1	192.9 ± 3.7*	188.7 ± 1.3*

Results are expressed as mean ± SEM. * p < 0.05 vs. respective control. n = 8–10 in each experimental group

### TNF-α reduces vascular relaxation

**Fig. 2:**
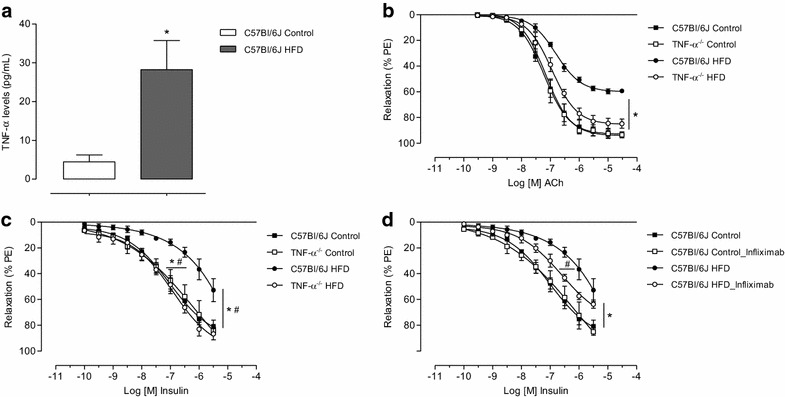
TNF-α decreases vascular relaxation in HFD-fed mice. Plasma TNF-α levels (**a**). Concentration-effect curves to acetylcholine and insulin were performed in endothelium-intact mesenteric resistance arteries of C57Bl/6J and TNF-α KO mice fed with control or HFD diets (**b**, **c**). The role of TNF-α on the vasculature was investigated using infliximab in vessels of C57Bl/6J fed with control or HFD diet (**d**). Results represent the mean ± S.E.M. n = 5–6 in each experimental group. *p < 0.05 vs. C57Bl/6J Control; ^#^p < 0.05 vs. C57Bl/6J HFD

As shown in Fig. 2a HFD-fed C57Bl/6J mice exhibited a 6.5-fold increase in plasma TNF-α levels compared with control mice. Figure 2b–d and Table 2 show that TNF-α contributes to reduced acetylcholine and insulin-induced vasodilation in HFD-fed mice. No difference was observed in vasodilation between C57Bl/6J and TNF-α KO mice fed with control diet. HFD reduced acetylcholine and insulin-induced vascular relaxation in C57Bl/6J mice. However, TNF-α deletion prevented HFD-induced vascular dysfunction (Fig. 2b, c). Endothelium removal abolished insulin-induced vasodilation in all groups. In addition, no significant differences were observed in relaxation mediated by sodium nitroprusside between wild-type and TNF-α KO mice or between control and HFD mice (not shown).

**Table 2:** *p*D_2_ and Emax (%) values of acetylcholine and insulin-induced relaxation in mesenteric arteries of control or HFD-fed mice incubated with vehicle or infliximab

Groups	*p*D_2_		Emax	
	Control	HFD	Control	HFD
C57Bl/6J (acetylcholine)	7.29 ± 0.06 (n = 6)	6.80 ± 0.04 (n = 6)*	92.8 ± 1.9 (n = 6)	59.9 ± 1.8 (n = 6)*
TNF-α^−/−^ (acetylcholine)	7.16 ± 0.02 (n = 6)	6.94 ± 0.04 (n = 6)^#^	94.4 ± 2.1 (n = 6)	87.8 ± 1.3 (n = 6)^#^
C57Bl/6J (insulin)	7.01 ± 0.15 (n = 5)	6.02 ± 0.18 (n = 6)*	80.8 ± 2.7 (n = 5)	52.8 ± 6.8 (n = 5)*
TNF-α^−/−^ (insulin)	6.84 ± 0.51 (n = 5)	6.91 ± 0.20 (n = 6)^#^	84.4 ± 2.1 (n = 5)	86.7 ± 2.9 (n = 5)^#^
C57Bl/6J_Infliximab	7.03 ± 0.14 (n = 5)	6.69 ± 0.21 (n = 6)^#^	85.0 ± 1.8 (n = 5)	63.7 ± 2.2 (n = 5)*

Data represent the mean ± SEM of n experiments. Two-way ANOVA with Bonferroni post-test. * p < 0.05 vs. C57Bl/6J Control; ^#^ p < 0.05 vs. C57Bl/6J HFD

To assess direct effects of TNF-α in the vasculature, vessels were incubated with infliximab, a chimeric monoclonal antibody against TNF-α. Infliximab did not affect insulin-induced vascular relaxation in C57Bl/6J mice fed with the control diet. However, infliximab augmented insulin vasodilation in HFD-fed C57Bl/6J mice (Fig. 2d).

### TNF-α and PTEN-dependent mechanisms contribute to vascular insulin resistance in HFD-fed mice

**Fig. 3:**
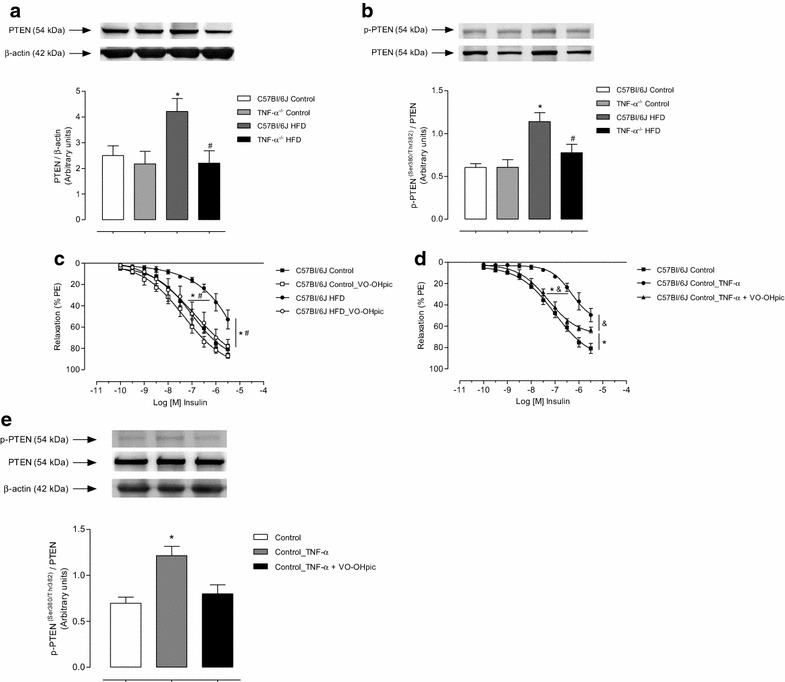
Vascular PTEN protein phosphorylation modulates insulin-induced relaxation in HFD-fed mice. Western blot quantification of total (**a**) and phosphorylated (**b**) PTEN expression levels in mesenteric arteries. Concentration-effect curves to insulin were performed in endothelium-intact resistance mesenteric arteries. The role of PTEN in the vasculature was investigated using VO-OHpic in vessels of C57Bl/6J mice fed with control and HFD diets (**c**) and vessels of C57Bl/6J incubated with TNF-α (**d**). Western blot quantification of phosphorylated PTEN expression levels in mesenteric arteries (**e**). Results represent the mean ± S.E.M. n = 5–6 in each experimental group. *p < 0.05 vs. C57Bl/6J Control; ^#^p < 0.05 vs. C57Bl/6J HFD; ^&^p < 0.05 vs. C57Bl/6J Control_TNF-α

Figure 3a, b illustrates total and phosphorylated levels of PTEN in the mesenteric bed of the experimental groups. HFD treatment increased vascular PTEN protein expression, as well as PTEN phosphorylation levels in C57Bl/6J mice, effects not seen in vessels from TNF-α KO mice. To assess whether PTEN is involved in HFD-induced vascular insulin resistance, mesenteric vessels from C57Bl/6J mice, fed with control or HFD, were incubated with a PTEN inhibitor (VO-OHpic) (Fig. 3c; Table 3). The PTEN inhibitor did not affect relaxation in arteries from control mice. However, VO-OHpic completely prevented vascular insulin resistance in vessels from HFD-fed C57Bl/6J mice. In addition, control vessels incubated with recombinant TNF-α exhibited decreased insulin-induced relaxation. Concomitant incubation of vessels with TNF-α and the PTEN inhibitor prevented the effects of TNF-α on insulin vasodilation (Fig. 3d; Table 4). In this context, TNF-α increased PTEN phosphorylation, which was reversed in the presence of PTEN inhibitor (Fig. 3e).

**Table 3:** *p*D_2_ and Emax (%) values of insulin-induced relaxation in mesenteric arteries of control or HFD-fed mice incubated with vehicle, VO-OHpic or TNF-α

Groups	*p*D_2_		Emax	
	Control	HFD	Control	HFD
C57Bl/6J	7.01 ± 0.15 (n = 5)	6.02 ± 0.18 (n = 6)*	80.8 ± 2.7 (n = 5)	52.8 ± 6.8 (n = 5)*
C57Bl/6J_VO-OHpic	6.93 ± 0.22 (n = 5)	6.87 ± 0.11 (n = 7)^#^	86.9 ± 1.9 (n = 5)	78.0 ± 4.1 (n = 5)^#^
C57Bl/6J_TNF-α	6.30 ± 0.14 (n = 6)*	–	49.4 ± 2.7 (n = 6)	–
C57Bl/6J_TNF-α + VO-OHpic	7.29 ± 0.13 (n = 6)^&^	–	63.5 ± 1.6 (n = 6)*^&^	–

Data represent the mean ± SEM of n experiments. Two-way ANOVA with Bonferroni post-test. * p < 0.05 vs. C57Bl/6J Control; ^#^ p < 0.05 vs. C57Bl/6J HFD; ^&^ p < 0.05 vs. C57Bl/6J_TNFα

**Table 4:** *p*D_2_ and Emax (%) values of insulin-induced relaxation in mesenteric arteries of control or HFD-fed mice incubated with vehicle, VO-OHpic, TNF-α or L-NAME

Groups	*p*D_2_		Emax	
	Control	HFD	Control	HFD
C57Bl/6J	7.01 ± 0.15 (n = 5)	6.02 ± 0.18 (n = 6)*	80.8 ± 2.7 (n = 5)	52.8 ± 6.8 (n = 5)*
C57Bl/6J_YS-49	6.99 ± 0.19 (n = 5)	7.12 ± 0.10 (n = 6)^#^	91.8 ± 1.3 (n = 5)*	83.7 ± 2.0 (n = 5)^#^
C57Bl/6J_L-NAME	6.30 ± 0.14 (n = 6)*	–	47.4 ± 4.7 (n = 6)*	–
C57Bl/6J_TNF-α + VO-OHpic	7.29 ± 0.13 (n = 6)	–	63.5 ± 1.6 (n = 6)	–
C57Bl/6J_TNF-α + VO-OHpic + L-NAME	6.49 ± 0.23 (n = 6)^&^	–	33.5 ± 5.6 (n = 6)^&^	–

Date represent the mean ± SEM of n experiments. Two-way ANOVA with Bonferroni post-test. * p < 0.05 vs. C57Bl/6J Control; ^#^ p < 0.05 vs. C57Bl/6J HFD; ^&^ p < 0.05 vs. C57Bl/6J_TNFα + VO-OHpic

### HFD-induced obesity impairs Akt/NO signaling pathway by TNF-α-dependent mechanisms

**Fig. 4:**
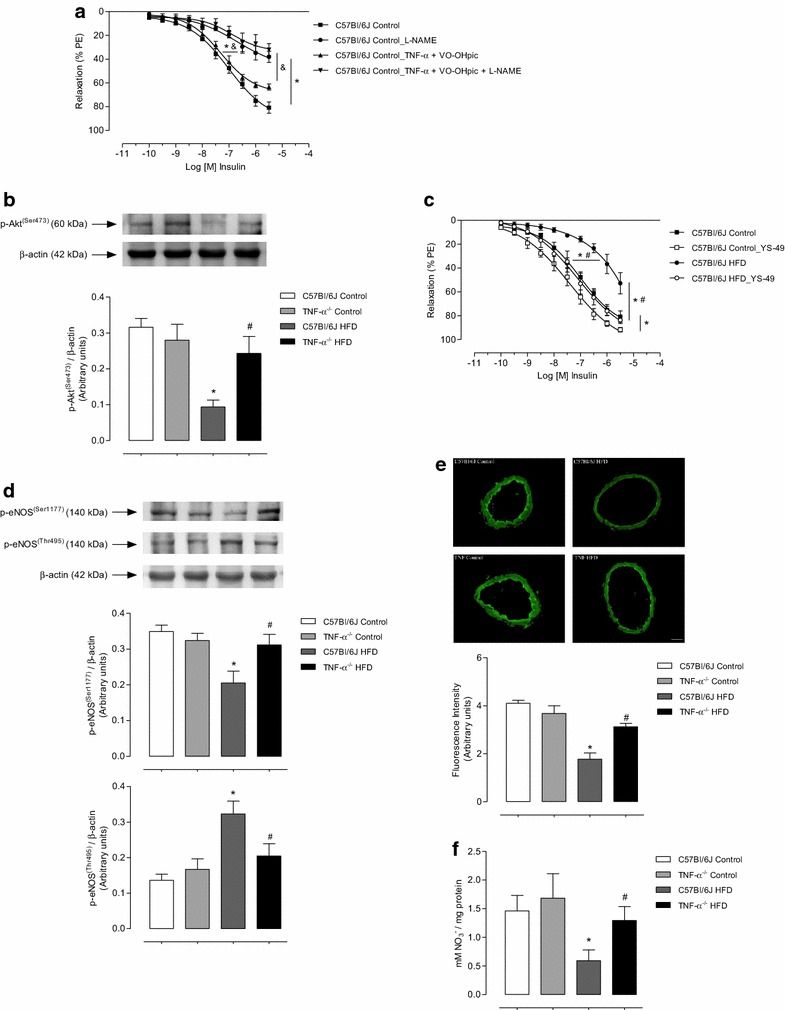
TNF-α contributes to decreased Akt/NO signaling in HFD-fed mice. Concentration-effect curves to insulin were performed in endothelium-intact mesenteric arteries. The role of the PTEN on TNF-α-modulate NO in vessels was investigated using L-NAME (**a**). Western blot quantification of Akt^(Ser473)^ phosphorylation levels in mesenteric arteries (**b**). The role of Akt on the relaxation was investigated using YS-49 in vessels of C57Bl/6J mice fed with control or HFD diet (**c**). Western blot quantification of mesenteric arteries eNOS^(Ser1177/Thr495)^ phosphorylation levels in mesenteric arteries (**d**). DAF-2 DA-derived fluorescence (**e**). NO metabolites levels (**f**). Results represent the mean ± S.E.M. n = 5–6 in each experimental group. Scale bar: 100 µm. *p < 0.05 vs. C57Bl/6J Control; ^#^p < 0.05 vs. C57Bl/6J HFD; ^&^p < 0.05 vs. C57Bl/6J_TNFα + VO-OHpic

In order to elucidate the mechanisms involved on the crosstalk between TNF-α and PTEN, which affects the sensitivity of mesenteric arteries to insulin, the role of PTEN on the modulatory effects of TNF-α in vascular NO bioavailability was evaluated. The nitric oxide synthase inhibitor L-NAME significantly reduced insulin vasodilation in C57Bl/6J mice on control diet. L-NAME also effectively reduced insulin-induced relaxation in vessels incubated with TNF-α and PTEN inhibitor (Fig. 4a; Table 4), indicating that PTEN activity is involved on TNF-α-induced decreased NO bioavailability in mesenteric arteries.

As TNF-α increases PTEN activity, reducing Akt activity [21] and NO release, we investigated whether TNF-α decreases insulin-induced vascular relaxation by interfering with Akt/NO signaling in HFD-fed mice.

The vasculature of HFD-fed C57Bl/6J mice exhibited reduced phosphorylation levels of Akt (Ser^473^), which was not observed in vessels from HFD-fed TNF-α KO mice (Fig. 4b). To determine whether Akt signaling pathway is involved on insulin vascular resistance in HFD-fed mice, vessels were pre-incubated with an Akt activator, YS-49, prior to the concentration-effect curves to insulin (Fig. 4c; Table 4). The Akt activator restored insulin relaxation in mesenteric arteries from HFD-fed C57Bl/6J mice. YS-49 did not change insulin-induced vascular relaxation in mice fed with the control diet.

Signaling downstream to Akt, i.e. phosphorylation levels of eNOS (at Ser^1177^ and Thr^495^, stimulatory and inhibitory sites, respectively), was also analyzed (Fig. 4d). Vessels from HFD-fed C57Bl/6J mice presented reduced levels of phosphorylated eNOS at Ser^1177^ residue and increased levels of phosphorylated eNOS at Thr^495^ residue. Vessels from HFD-fed TNF-α receptor deficient mice exhibited increased Ser^1177^ phosphorylation levels and decreased Thr^495^ phosphorylation.

To confirm that reduced vascular eNOS (Ser^1177^) phosphorylation levels in HFD-fed C57Bl/6J mice are associated with reduced NO release, NO levels were determined by using two techniques: the fluorescent NO indicator (DAF-2 DA) (Fig. 4e) and measurement of NO metabolites levels (Fig. 4f). The vasculature of HFD-fed C57Bl/6J mice exhibited decreased NO formation, which was not observed in vessels from HFD-fed TNF-α KO mice.

## Discussion

Obesity induces various structural and functional changes in the vasculature, compromising the integrity and function of the cardiovascular system. The present study investigated mechanisms by which TNF-α decreases vascular insulin sensitivity consequent to a HFD. The key finding of our study is that HFD increases TNF-α circulating levels, which increases PTEN phosphatase activity, impairing Akt/eNOS/NO signaling pathway and compromising vascular relaxation.

### High fat diet and TNF-α

Obesity development is usually associated with consumption of diets rich in fat and carbohydrates [29]. Increased body mass associated with fat accumulation is an important criterion for obesity characterization [30]. In the present study HFD significantly increased adiposity index and triggered the accumulation of adipose tissue in C57Bl/6J mice. These conditions facilitate development of co-morbidities that result in increased glucose levels and body mass. Of importance, TNF-α KO mice fed with HFD for 18 weeks also showed increased body weight, increased adiposity index and serum glucose levels, indicating that the differences found in vascular function between C57Bl/6J and TNF-α KO mice do not depend on the improvement of body mass profile or body composition phenotype.

Obesity is also characterized by the development of a chronic low-grade inflammation. The concept of metabolic inflammation came from the identification of high levels of circulating TNF-α, an inflammatory cytokine associated with lipid [31] and carbohydrates [32] metabolism. Accordingly, HFD-fed C57Bl/6J mice exhibited increased TNF-α plasma levels. TNF-α and other cytokines have been described as biomarkers of cardiovascular risk in metabolic diseases. For instance, patients with type 2 diabetes mellitus and hypertension show severe increase of serum TNF-α [33], which is significantly attenuated by acetylsalicylic acid [34]. In this context, TNF-α blockade has antiatherosclerotic effects in hyperlipidemic mice lacking apoE [35].

High fat intake is associated with major risk factors for development of type 2 diabetes mellitus and cardiovascular disorders [36]. Mice fed with HFD exhibited glucose tolerance and increased plasma levels of insulin. The lack of TNF-α receptors partially protected against these metabolic abnormalities, in line with previous reports. Uysal (1997) demonstrated that TNF-α deficient mice are protected against HFD-induced increased glucose and insulin levels, but not body weight gain [37]. In addition, TNF-α or TNF-α receptors deficient mice are protected against obesity-induced insulin resistance and hyperglycemia [38, 39]. Furthermore, TNF-α blocks glucose uptake by inhibiting insulin-stimulated tyrosine kinase activity of the insulin receptor in various cell types, including adipocytes, hepatocytes and muscle cells [40].

### TNF-α and vascular insulin resistance

Insulin directly contributes to vascular tone control [41–43]. Insulin stimulates NO production by mechanisms involving activation of PI3K/Akt/eNOS signaling in the endothelial layer. Insulin-induced vasodilation increases blood flow and stimulates glucose uptake by the skeletal muscle, thereby linking metabolic and hemodynamic homeostasis [44].

Vascular insulin resistance is characterized by the inability of a tissue to respond to insulin [45] and vascular insulin resistance is associated with decreased NO production. In this study HFD promoted insulin resistance in the vasculature and deletion of TNF-α receptors prevented this effect. TNF-α is involved in endothelial dysfunction and reduced NO release in obese individuals [46, 47]. TNF-α also favors release of contractile mediators [48], such as COX-2-derived products and reactive oxygen species (ROS) [49]. Of importance, endothelial dysfunction evoked by TNF-α is reduced in fit and well-controlled type 1 diabetes mellitus patients [50]. In addition, TNF-α plays an important role in the changes of macrovascular and microvascular circulation. Oleate, the main lipid component of virgin olive oil, protects against cardiovascular insulin resistance and improves endothelial dysfunction in response to TNF-α [51].

An important finding of this study is that infliximab increased insulin sensitivity of mesenteric arteries from HFD-fed C57Bl/6J mice, indicating a synergic effect between TNF-α produced by the vasculature and circulating TNF-α. Infliximab treatment has anti-inflammatory effects in the vasculature and improves endothelium-dependent vasomotor responses in patients with systemic vasculitis [52].

### Crosstalk between TNF-α, PTEN and vascular dysfunction

It is well known that TNF-α interferes with vascular beneficial effects of insulin, possibly at the level of IRS [53]. Our results show the involvement of PTEN on TNF-α-induced reduced vascular insulin responses. There is evidence showing that PTEN modulates hyperglycemia and insulin resistance [54]. In addition, PTEN deletion in pancreatic α-cells protects against HFD-induced insulin resistance [55]. PTEN, through its lipid phosphatase activity, catalyzes the conversion of PIP3 (Akt substrate) to PIP2 by dephosphorylating the 3-position of the inositol ring of PIP3, attenuating Akt/eNOS/NO signaling [56].

PTEN expression changes under different conditions. PTEN mRNA decreases in the adipose tissue after exposure to cold, but increases with obesity [57]. The present study shows that HFD-fed C57Bl/6J mice exhibit increased vascular expression and activity of this phosphatase. Of importance, TNF-α receptor deletion prevented HFD-induced increased PTEN expression and activity. Experimental evidence indicates that NF-kappaB signaling pathway is the link between TNF-α and PTEN in leukemic [22], glioma [58] and intestinal cells [59]. Mechanisms linking TNF-α receptor and PTEN activation/expression were not investigated, which represents a limitation of our study.

One of the mechanisms that regulate PTEN activity is reversible oxidation of the cysteine residue at the phosphatase active site [60]. Accordingly, treatment of macrophages with lipopolysaccharide stimulates ROS production and increases the fraction of oxidized PTEN from 5 to 16 % [61], showing a crosstalk between ROS production by the immune system and PTEN regulation.

In hepatocytes TNF-α increases PTEN expression, which is blunted by PTEN siRNA knockdown and VO-OHpic treatment [62]. These results suggest an important crosstalk between inflammatory mediators and PTEN activity and are in line with the idea that PTEN is involved in apoptosis and inflammatory processes [18]. Our findings indicate that TNF-α is a positive modulator of PTEN activity. Since PTEN inhibition restores vascular insulin sensitivity, decreased by TNF-α and HFD, PTEN may be considered a major contributor to TNF-α-induced insulin resistance.

The mechanisms whereby PTEN changes vascular function are poorly understood. Our data indicate that TNF-α via increased PTEN expression and activity compromises NO bioavailability. Accordingly, mesenteric arteries incubated with TNF-α present reduced sensitivity to insulin effects as well as reduced Akt/eNOS signaling and NO levels. PTEN inhibition improved insulin-dependent vasodilation, and restored NO levels, as indicated by the effects of L-NAME on insulin-induced vascular relaxation. In addition, TNF-α is the major regulator of PTEN activity in the vasculature of HFD-fed mice, decreasing Akt/eNOS/NO signaling. Our findings corroborate a previous report showing decreased NO release by human aortic endothelial cells overexpressing PTEN [63]. On the other hand, NO by inducing S-nitrosylation and ubiquitination, modulates both PTEN protein degradation and enzymatic activity in neurons, representing a regulatory mechanism of the Akt/NOS signaling pathway on PTEN [64].

## Conclusions

Taken together, our study suggests that in obesity, TNF-α induces vascular insulin resistance by increasing PTEN activity that negatively modulates Akt/eNOS/NO signaling and insulin vasodilation. Since vascular insulin resistance represents a primary defect in vascular dysfunction, TNF-α and PTEN are potential therapeutic targets for obesity-associated cardiovascular and metabolic dysfunction.

## Abbreviations

PI3K: phosphatidylinositol 3-kinase
eNOS: endothelial nitric oxide synthase
NO: nitric oxide
HFD: high fat diet
TNF-α: tumor necrosis factor alpha
PTEN: phosphatase and tension homologue
